# RNA editing in host lncRNAs as potential modulator in SARS-CoV-2 variants-host immune response dynamics

**DOI:** 10.1016/j.isci.2024.109846

**Published:** 2024-04-29

**Authors:** Partha Chattopadhyay, Priyanka Mehta, Kanika, Pallavi Mishra, Chinky Shiu Chen Liu, Bansidhar Tarai, Sandeep Budhiraja, Rajesh Pandey

**Affiliations:** 1 Division of Immunology and Infectious Disease Biology, INtegrative GENomics of HOst-PathogEn (INGEN-HOPE) laboratory, CSIR-Institute of Genomics and Integrative Biology (CSIR-IGIB), Mall Road, Delhi 110007, India; 2 Academy of Scientific and Innovative Research (AcSIR), Ghaziabad 201002, India; 3 Max Super Speciality Hospital (A Unit of Devki Devi Foundation), Max Healthcare, Delhi 110017, India

**Keywords:** Biological sciences, Molecular biology, Immune response, Virology

## Abstract

Both host and viral RNA editing plays a crucial role in host’s response to infection, yet our understanding of host RNA editing remains limited. In this study of in-house generated RNA sequencing (RNA-seq) data of 211 hospitalized COVID-19 patients with PreVOC, Delta, and Omicron variants, we observed a significant differential editing frequency and patterns in long non-coding RNAs (lncRNAs), with Delta group displaying lower RNA editing compared to PreVOC/Omicron patients. Notably, multiple transcripts of *UGDH-AS1* and *NEAT1* exhibited high editing frequencies. Expression of *ADAR1/APOBEC3A/APOBEC3G* and differential abundance of repeats were possible modulators of differential editing across patient groups. We observed a shift in crucial infection-related pathways wherein the pathways were downregulated in Delta compared to PreVOC and Omicron. Our genomics-based evidence suggests that lncRNA editing influences stability, miRNA binding, and expression of both lncRNA and target genes. Overall, the study highlights the role of lncRNAs and how editing within host lncRNAs modulates the disease severity.

## Highlights

• Editing of host lncRNA in Delta infected patients differs from PreVOC and Omicron
• Repeat element abundance is a plausible modulator of RNA editing
• Editing in lncRNA has implications of infection associated biological functions
• Editing may affect lncRNA stability, miRNA/mRNA interactions and gene expression

## Graphical abstract

**Figure:**
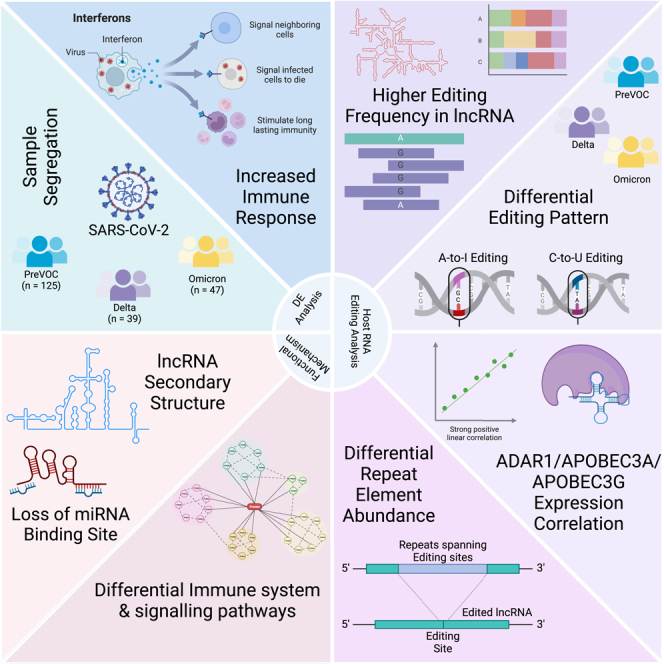

## Introduction

The alteration of genetic information at the level of RNA is a crucial epi-transcriptomic modification with the potential to impact many biological functions. RNA editing is one such epi-transcriptional modification wherein the RNA transcript sequence deviates from the corresponding DNA sequence due to nucleotide base modification.^1^^,^^2^ Adenosine and cytidine deaminases are the two primary and crucial players in the RNA editing events that catalyze the deamination of adenosine and cytosine residues in the RNA moieties to inosine and uracil residues, respectively. Such editing events generate diverse transcripts within identical cell populations, contributing to heterogeneity and phenotypic plasticity. The A-to-I (decoded as G) editing happens via the ADAR (adenosine deaminases acting on RNA) enzymes that specifically work on double-stranded RNA (dsRNA). In mammals, four genes expressing three isoforms of ADARs have been reported including *ADAR1* (*ADAR1p150* and *ADAR1p110*), *ADAR2*, and *ADAR3*. Globally, *ADAR1* is seen to perform its editing functions in the repetitive regions such as Alu sites. *ADAR2*, on the other hand, primarily performs RNA editing in the non-repetitive regions of the coding sites. *ADAR3*, however, does not act as an RNA editor and has been shown to inhibit RNA editing. Majorly, editing occurs in the non-coding RNAs, with only a very small percentage of RNA editing taking place in the protein coding RNAs.^3^^,^^4^

C-to-U editing occurs via the APOBEC (apolipoprotein B editing complex) family of RNA editing enzymes. Ten isoforms of APOBEC proteins are expressed in humans including APOBEC1, APOBEC2, APOBEC3A, APOBEC3B, APOBEC3C APOBEC3D, APOBEC3F, APOBEC3G, APOBEC3H, and APOBEC4. APOBEC1 has been shown to preferentially target AU-rich regions present in the 3′UTRs of protein-coding RNAs. Viral elements or RNAs are targeted by the APOBEC3 isoforms. On the other hand, genomes and transcriptomes of infecting RNA viruses are directly impacted because of RNA editing, creating nucleotide diversity in the viral genome. Multiple reports have highlighted the role of RNA editing in creating single nucleotide variants (SNVs) via substitution mutations.^5^ Kurkowiak et al. reported the involvement of C to U editing of severe acute respiratory syndrome coronavirus 2 (SARS-CoV-2) in enhancing genetic diversity, thus creating evolutionary adaptation possibilities.^6^ Another report by Crooke III et al. demonstrated decreased A-to-I editing in the host Alu RNAs after SARS-CoV-2 infection. These unedited Alu RNA elements have been known to induce inflammatory response in the host, indicating decreased A-to-I editing as a key player in mediating host response during the infection.^7^ As such, RNA editing has been reported not only in primates, but also in plants, as well as lower organisms. Li et el. reported a high A-to-I editing in leaf-cutting ant *Acromyrmex echinatior*.^8^ Similarly, Liscovitch-Brauer et al. reported a high A-to-I editing in cephalopods.^9^ On the other side, Chu and Wei et al., and Duan et al. reported a high C-to-U editing in plants, with an important role in insect-plant interaction.^10^^,^^11^ In humans, the primary RNA editing event is the A-to-I editing, and nearly ∼97% of the editing sites are present within the repeat elements, especially Alu,^12^ emphasizing the role of understanding the dynamics of RNA editing in hosts.

RNA editing has been shown to play a crucial role in regulating the innate immune response and infection outcome. Viral dsRNA molecules during viral infection are detected and edited by RNA editors, thus helping in protection from dsRNA sensing molecules. This further helps in the evasion from the host’s innate immune response. ADAR1 has been shown to play both the proviral and antiviral roles by editing the viral RNA molecules. Edited viral RNA molecules impact the morphogenesis of the virus, thus altering cellular susceptibility to infection. Edited dsRNA has been shown to dodge innate immunity via (1) suppressing interferon response by depleting the target (unedited dsRNA) of MAVS (mitochondrial antiviral signaling) protein, (2) inhibiting protein kinase R (PKR), thus evading translational inhibition, and (3) avoiding the activation of RNaseL responsible for cleaving cellular and viral RNAs, preventing cellular apoptosis.^13^^,^^14^^,^^15^ The antiviral role of RNA editing is mediated by mRNA editing, thus creating faulty transcripts.^16^^,^^17^ Recently, an elevated C-to-U editing, speculated to be mediated by the APOBEC enzymes has been reported in SARS-CoV-2 genome, suggesting the crucial role of RNA editing in predicting infection outcome.^18^^,^^19^

Majority of studies report host mediated RNA editing in the infecting virus genetic material, promoting either antiviral or proviral effects.^5^^,^^20^^,^^21^^,^^22^^,^^23^ However, there is little evidence regarding the role of host RNA editing during an infection.^24^ The RNA editing events in the host may be essential for regulating the immune response leading to host defense since there is indirect evidence of pathogen induced upregulation of RNA editors and increased editing sites during an infection.^25^^,^^26^ The diverse effects of RNA editing include alteration of protein coding sequences by incorporation of start/stop codons, alteration of alternative splicing by modifying the splice sites, change in secondary structure, stability, and expression of both mRNAs and lncRNAs.^2^ Although lncRNAs possess a higher abundance of repeat elements and dynamic secondary structures, making them ideal candidates for RNA editing, there is limited information available regarding RNA editing in the lncRNAs. Qiu et al. have investigated the role of RNA editing in the lncRNA during early embryo development.^27^ Picardi et al. and Silvestris et al. reported identification of RNA editing sites within the lncRNAs in different forms of cancer,^28^^,^^29^ while Gong et al. have created LNCediting, a database for functional role of RNA editing in lncRNAs.^30^ Many studies, including from our group, have reported lncRNAs as a modulator of infectious disease severity. For example, lncRNA *ROR1-AS1* and *UGDH-AS1* have been reported to modulate immune response in the COVID-19 patients while *NEAT1*, *MALAT1*, and *LINC00273* have been reported to modulate both immune and inflammatory response in severe COVID-19 patients.^31^^,^^32^^,^^33^^,^^34^ Some of these lncRNAs, such as *NEAT1*, *MALAT1*, and *UGDH-AS1* have been reported to regulate the pathophysiology and outcomes in other infectious diseases (such as influenza A virus, dengue virus, human immunodeficiency virus) as well.^35^^,^^36^^,^^37^ Gaining insights into the host RNA editing would provide a clearer picture of the interplay between the host and the pathogen during an infection. The information obtained will be crucial for understanding host-pathogen interactions in a greater depth, and in turn combating the infection in a more robust way. Therefore, in this study, we delved into the investigation of potential differential RNA editing within the host lncRNAs across the three SARS-CoV-2 variants: PreVOC, Delta, and Omicron. Our findings reveal distinct RNA editing patterns within the lncRNAs in all three variants of concern, influencing crucial biological pathways relevant to infectious diseases. Through functional analysis of the in-house generated RNA sequencing (RNA-seq) data of the hospital admitted patients, we identify *ADAR1/APOBEC3* expression and repeat element abundance as key regulators of differential editing, and additionally provide genomic mechanistic evidence, including RNA secondary structure and miRNA binding site analysis, elucidating the functional role of RNA editing.

## Results

### Patient cohort characterization: Classification and clinical Evaluation

**Figure 1:**
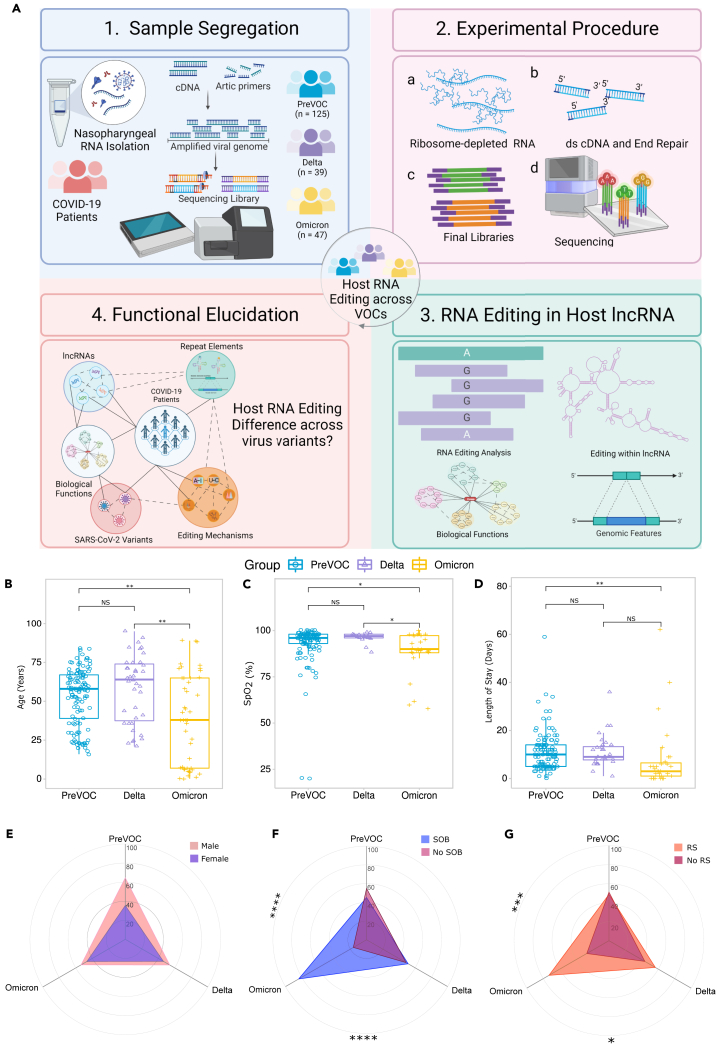
Study design, experimental workflow, and clinical data of the SARS-CoV-2 infected individuals across variants (A) Study design and experimental workflow including the segregation of samples into the SARS-CoV-2 variants, whole transcriptome sequencing of the patients, identification of RNA editing events within the host lncRNAs and functional understanding of the RNA editing events. (B–G) Clinical and demographic parameters of the patients. (B) Age (in years) of the patients, (C) SpO_2_ (%) levels of the patients, (D) length of hospital stay (in days), (E) gender distribution, (F) shortness of breath (SOB), and (G) requirement of respiratory support (RS) across the virus variants. The Mann-Whitney U test was applied for comparing age, SpO2, and length of stay across the groups, while Chi-square test was used for gender distribution, SOB and RS comparison across the groups. Significance value is denoted as ∗, where ∗ indicates *p* ≤ 0.05, ∗∗ indicates *p* ≤ 0.01, ∗∗∗ indicates *p* ≤ 0.001 and ∗∗∗∗ indicates *p* ≤ 0.0001.

We included 211 COVID-19 patients who were hospitalized to a tertiary care center between April 2020 and March 2022 in order to understand the possible regulatory function of host RNA editing as a modulator of disease pathology and if the editing differs across various strains of the same pathogen (SARS-CoV-2). The samples were collected from individuals who reported to the hospital for the first time after experiencing symptoms and were subsequently confirmed of SARS-CoV-2 infection, post RT-PCR and whole genome sequencing of the pathogen for variants conformation. These same samples were thereafter utilized in this retrospective study. They were segregated into three groups: PreVOC (*n* = 125), Delta (*n* = 39), and Omicron (*n* = 47) based on the pathogen’s in-house genome sequencing as part of genomic surveillance. The clinical and demographic data of the patients show median age (years), SpO_2_, and duration of hospital stay to be significantly lower in the Omicron patients compared to the PreVOC and Delta, while there was no significant difference in the gender distribution across the groups (Figures 1B–1E). Interestingly, a higher percentage of Omicron patients experienced breathing difficulties and subsequently required respiratory support compared to the PreVOC and Delta despite shorter hospital stay and milder symptoms (Figures 1F and 1G). Detailed clinical and demographic information as well as patient groupings are available in Table 1 and Table S1. The detailed study design has been depicted in Figure S1.

**Table 1:** Demographic and clinical parameters of the patients

	PreVOC	Delta	Omicron	PreVOC vs. Delta	Delta vs. Omicron	PreVOC vs. Omicron
Number	125	39	47			
Gender (M/F%)	35.2/64	46.2/53.8	42.6/51	0.1481^a^	0.8855^a^	0.1864^a^
Age range (years)	16–84	21–95	0.2–89	0.0846^b^	0.006^b^	0.0255^b^
CT value (RdRp gene)	25.16 ± 0.5	16.07 ± 0.5	18.04 ± 1.3	<0.0001^b^	0.1915^b^	0.0001^b^
SpO2 (%)	92.01 ± 1.4	96.13 ± 0.7	91.33 ± 1.9	0.3496^b^	0.1356^b^	0.2978^b^
Recovered (%)	85.6	41	59.6	<0.0001^a^	0.022^a^	<0.0001^a^
Respiratory support (Yes/No)	61/64	22/3	9/3	0.0003^a^	0.3666^a^	0.1292^a^
Hospital stay (days)	11.17 ± 0.7	3.94 ± 1.1	9.06 ± 2.6	<0.0001^b^	0.0737^b^	0.0288^b^
CRP	NA	28.72 ± 7.2	145.99 ± 28.4	NA	<0.0001^b^	NA
Fever	72.8	68.75	19.04	0.5331^a^	<0.0001^a^	<0.0001^a^
Cough	46.4	50	33.3	0.5713^a^	0.0147^a^	0.0601^a^
Shortness of breath	45.1	51.2	83.7	0.3958^a^	<0.0001^a^	<0.0001^a^
Diabetes	33.3	NA	66.6	NA	NA	<0.0001^a^
Hypertension	44.4	NA	50	NA	NA	0.3953^a^
Thyroid disorder	13.7	NA	64.3	NA	NA	<0.0001^a^

NA: data not available.

a Chi-square test.

b Mann-Whitney test.

### Differential immune and antiviral response across the SARS-CoV-2 variants

**Figure 2:**
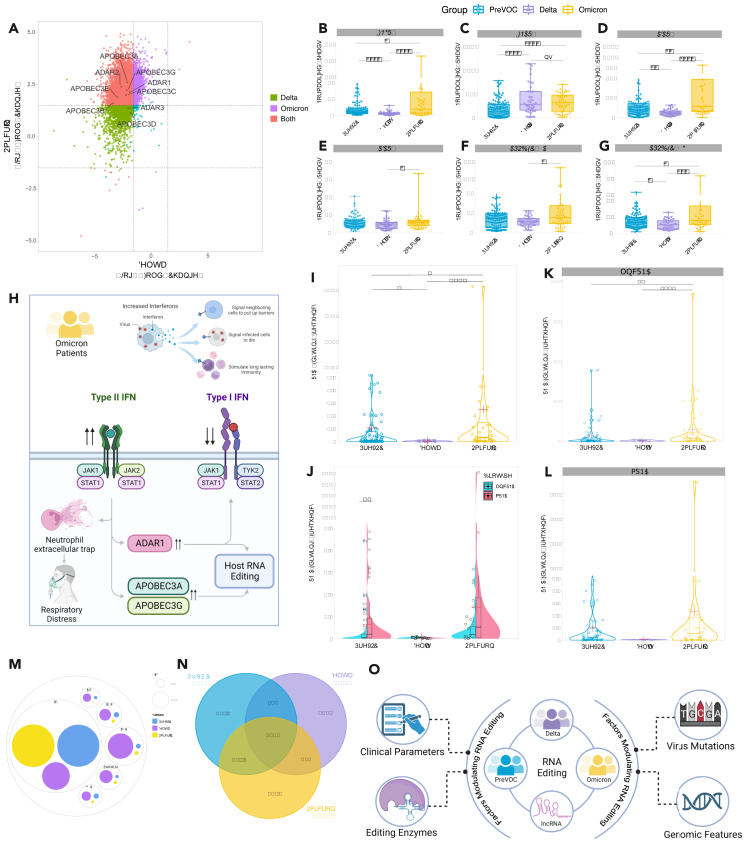
Differential host RNA editing pattern across the SARS-CoV-2 variants (A) Differential expression of genes between the PreVOC and Delta (on x axis) and the PreVOC and Omicron (on y axis). (B–G) Gene expression of (B) *IFNGR2*, (C) *IFNAR1*, (D) *ADAR1*, (E) *ADAR2*, (F) *APOBEC3A*, and (G) *APOBEC3G* across the SARS-CoV-2 variants. (H) Graphical illustration of type II IFN mediated regulation of *ADAR/APOBEC3* genes and its possible implications on type I IFN expression as well as respiratory distress in the Omicron patients. (I) Cumulative human host RNA editing frequency across the SARS-CoV-2 variants. (J) Differential RNA editing frequency between the lncRNAs and mRNAs. (K and L) (K) Differential RNA editing within the lncRNAs and (L) mRNAs across the virus variants. (M) Different types of editing within the lncRNAs across the groups. (N) Overlap between the number of lncRNA genes edited across the groups. (O) Graphical illustration of different factors that possibly modulate differential editing within the lncRNAs across the SARS-CoV-2 variants. The Mann-Whitney U test was applied for comparing gene expression and editing frequency across the groups. Significance value is denoted as ∗, where ∗ indicates *p* ≤ 0.05, ∗∗ indicates *p* ≤ 0.01, ∗∗∗ indicates *p* ≤ 0.001 and ∗∗∗∗ indicates *p* ≤ 0.0001.

We performed differential expression (DE) analysis of the genes across the groups (PreVOC vs. Delta, Delta vs. Omicron, and PreVOC vs. Omicron) for a comprehensive understanding of the differential host transcriptomic response to the different variants of SARS-CoV-2 (Figures S2A–2C). We observed a strong upregulation of interferon response in the Omicron group compared to the PreVOC and Delta (Figures S2D–S2F). The increased interferon response in Omicron patients has been reported in a few other studies as well.^38^^,^^39^^,^^40^ Consequently, the interferon stimulated gene (ISG) expression was also higher in the Omicron infected patients. Among the ISGs, the RNA editing related genes of *ADAR* and *APOBEC3* families were significantly upregulated in the Omicron but downregulated in the Delta, compared to the PreVOC group (Figure 2A). It is important to note that among the interferon family genes, only the interferon gamma receptor gene (*IFNGR2*) was upregulated in the Omicron while the interferon alpha receptor gene (*IFNAR1*) was significantly downregulated (Figures 2B and 2C). Among the ADAR family genes, *ADAR1* is known to be stimulated by interferon whereas *ADAR2* and *ADAR3* are constitutively expressed.^41^

The *ADAR1* expression was higher in the Omicron group compared to the PreVOC and Delta, while *ADAR2* and *ADAR3* expression was different compared to the Delta (Figures 2D and 2E). A similar trend in expression was observed for *APOBEC3A* and *APOBEC3G*, the two ISGs from the APOBEC3 family, while the expression of other APOBEC3 family genes did not differ significantly (Figures 2F and 2G). The expression profile of the *IFN*, *ADAR*, and *APOBEC3* genes could provide a possible explanation for the higher respiratory distress in the Omicron group. The Omicron infected patients had a higher expression of both type I and type II IFN. Multiple studies have also reported an increased IFN response in the Omicron group. *ADAR1* and *APOBEC3G* are stimulated by the increased IFN expression,^41^^,^^42^ and subsequently, *ADAR1* suppresses the expression of type I IFN while further increasing the expression of inflammatory genes as well as type II IFN,^43^^,^^44^^,^^45^ which on one side, increases the neutrophil extracellular trap (NET) formation, leading to increased respiratory distress,^46^^,^^47^ and on the other hand, further increase the expression of *ADAR1*, *APOBEC3A*, and *APOBEC3G*, which are IFN stimulated genes (Figure 2H). *ADAR2*, which is constitutively expressed, has a lower expression compared to *ADAR1*. *ADAR1*, apart from being the key editing enzyme, is also reported to target IFN type I gene to downregulate its expression.^43^^,^^44^ The DE profile of *ADAR1*, *APOBEC3A*, and *APOBEC3G* across the group also suggests differential RNA editing, which is a key component of the host antiviral response, across the studied patient groups.

### Host RNA editing is different across the SARS-CoV-2 variants

Since ADAR and APOBEC3 family members are involved in RNA editing, we then checked the host RNA editing across the groups using SPRINT, which is an unsupervised, *de-novo* SNP-free toolkit for identification of RNA editing sites from the RNA-seq reads.^48^ We observed significantly high cumulative RNA editing frequency in the Omicron patients compared to the PreVOC and Delta (Figure 2I). Further, the total editing events were queried for their presence within the lncRNAs and mRNAs, wherein the editing frequency between the mRNAs and lncRNAs was different only in the PreVOC group (Figures 2J; Tables S2A–S2F). Importantly, we observed differential editing frequencies across the groups only in case of the lncRNAs and not in the mRNAs (Figures 2K and 2L). *lnc-BICRAL-3:1* was among the highest edited lncRNA transcript, followed by *UGDH-AS1:10* and *lnc-NPIPB12-1:1* in the PreVOC group, whereas *lnc-FRG1-8:1*, *lnc-FRG1-8:2*, and *LINC01766:2* were among the highest edited lncRNA transcript in the Delta infected patients (Tables S2A–S2C). *lnc-NPIPB12-1:1*, *NEAT1:28*, and *NEAT1:27* were the top 3 edited transcripts in the Omicron patients, along with multiple other *NEAT1* transcripts bearing high number of editing sites (Tables S2A–S2C). We also observed a large number of editing sites within multiple transcripts of *UGDH-AS1* and *NEAT1* in both the PreVOC and in Omicron patients, but not in the Delta, where no edits were observed in *UGDH-AS1*, and only edited *NEAT1* transcript was *NEAT1:29*. We also cross-referenced all the RNA editing sites captured in our dataset with known RNA editing sites from REDIportal, which is the largest RNA editing resource for human and other organisms.^49^ It is important to note that while not all sites align, our analysis has successfully identified 40 sites in the lncRNA category and 169 sites in the mRNAs, which are consistent with known reported editing in the non-diseased human tissues. The limited overlap can be attributed to the specific context of our dataset, reflecting RNA editing patterns induced by viral infection. The different editing types, quantified in terms of percentage of RNA-DNA difference (RDD%) were also different across the PreVOC, Delta and Omicron, where the Delta patients have a significantly different editing pattern compared to the PreVOC and Omicron and A>G was found to be the most abundant RDD event (94.88% in the PreVOC, 40.91% in the Delta, and 97.68% in the Omicron group (Figure 2M). Moreover, the number of edited lncRNAs was also different across the groups, with a small overlap, emphasizing differential editing patterns across the three groups (Figure 2N).

### *ADAR/APOBEC3* expression and repeat element abundance modulate editing in host lncRNAs across the SARS-CoV-2 variants

**Figure 3:**
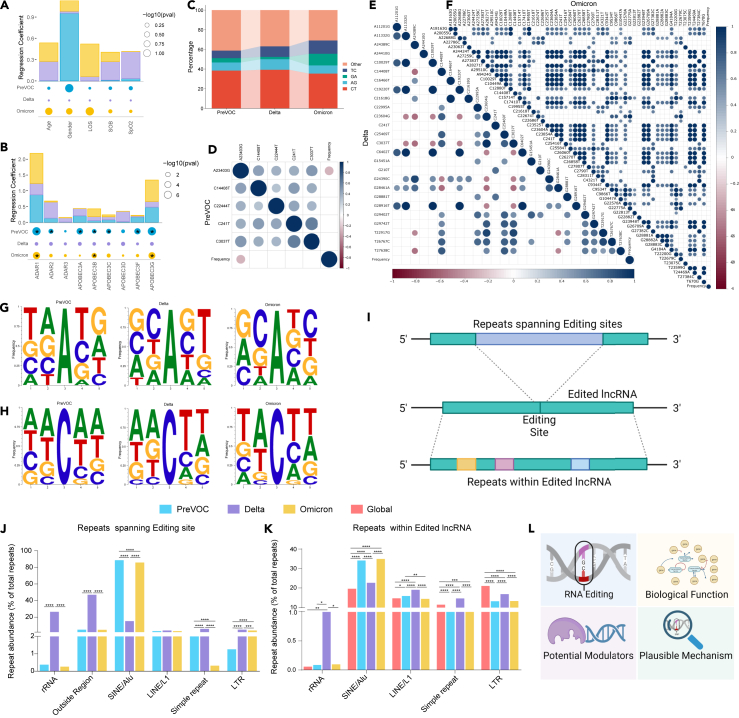
Factors modulating differential RNA editing across the virus variants (A and B) Linear regression analysis between (A) clinical parameters and editing frequency, (B) *ADAR/APOBEC3* expression and editing frequency. (C) Types of SNVs observed in the SARS-CoV-2 genomes across the virus variants. (D–F) Pearson correlation analysis of viral SNV and host lncRNA editing frequency in (D) PreVOC, (E) Delta, and (F) Omicron patients. (G and H) Bases adjacent to (G) A>G SNV sites, and (H) C>T SNV sites in the SARS-CoV-2 genome. (I) Graphical representation of the repeat element enrichment analysis at the editing site as well as cDNA of the edited lncRNA. (J) Repeat elements spanning the editing sites within the lncRNAs. The abundance was expressed as %age of the total repeats. (K) Repeat element abundance within the cDNA region of the edited lncRNAs. (L) Conceptual summary highlighting the potential factors modulating differential RNA editing across variants, leading to investigation of biological functions of the differential RNA editing and its potential mechanism. ANOVA was used to calculate the statistical significance of the linear regression model, while Chi-square test was used for comparison of repeat element distribution across groups. Significance value is denoted as ∗, where ∗ indicates *p* ≤ 0.05, ∗∗ indicates *p* ≤ 0.01, ∗∗∗ indicates *p* ≤ 0.001 and ∗∗∗∗ indicates *p* ≤ 0.0001.

The diverse editing patterns observed across the COVID-19 patient groups could stem from various factors, including differences in the clinical parameters, distinct expression profiles of editing enzymes, or potential impacts from the pathogen mutations and host genomic features (Figure 2O). To investigate whether clinical parameters might be correlated with RNA editing, we conducted linear regression analysis between the age/gender/length of hospital stay/shortness of breath/SpO_2_, and host RNA editing frequency; finding no significant association with any of the clinical parameters (Figure 3A). However, the immune/inflammatory response is a potential modulator of RNA editing, as the *ADAR1* and *APOBEC3G* genes are IFN stimulated genes. Therefore, we have checked for the expression of immune marker genes, and their correlation with the host RNA editing. We found a significant correlation between the expression of *IL1A/S100A8* and the editing frequency in the PreVOC group; however, no significant correlations were observed in the other two groups of Delta and Omicron (Figures S2G–S2I). Subsequently, to assess potential regulation of RNA editing by the *ADAR/APOBEC3* expression, linear regression analysis was performed between the *ADAR/APOBEC3* expression and the editing frequency. The aim of the linear regression analysis between *ADAR/APOBEC3* expression and editing frequency was to identify any potential differential association of the editing enzymes with the editing frequency, rather than determining ADAR and APOBEC3 as primary editing enzymes. This was done especially because of the observed differences in the editing pattern across the three variants. Notably, *ADAR1*, *APOBEC3A*, and *APOBEC3G* emerged as the key potential modulators of the host RNA editing in the PreVOC and Delta infected patients (Figure 3B). However, in the Delta, none of the editing enzymes exhibited a significant association with RNA editing, suggesting the potential involvement of another factor influencing the distinct RNA editing patterns observed in this group.

To explore the potential association between the SARS-CoV-2 mutations and host RNA editing, we initially identified SNVs within the SARS-CoV-2 genomes from the same set of samples across different groups. This was especially important, since multiple studies have reported specific SNVs to elicit different host responses. This is also the basis of the variant-specific differential host response during SARS-CoV-2 infection. A classic example of this is the spike D614G amino acid change caused by an A-to-G nucleotide mutation at position 23,403 in the Wuhan reference strain, leading to increased expression of interferon-stimulated genes (ISG).^50^ Broadly, we observed four different types of SNVs: C>T, A>G, G>A, and T>C across the SARS-CoV-2 variants (Figures 3C; Table S3). Unlike host RNA editing, C>T substitution was the predominant substitution type in the SARS-CoV-2 genome. We performed Pearson correlation analysis between the presence of any SNVs in the SARS-CoV-2 genome and the host RNA editing frequency. However, only one substitution (A23403G) showed a significant correlation with host RNA editing in the PreVOC group, and no significant correlation was observed between any other viral SNVs and host RNA editing (Figures 3D–3F). Notably, the flanking bases to the A>G SNV sites differed across the groups, while the flanking bases to the C>T SNV sites were primarily AA/TA/TT, indicating that the majority of the C>T SNVs may actually have resulted from APOBEC3-mediated cytosine to uracil modification (Figures 3G and 3H).

Given that the majority of editing sites are reported to reside within the repeat elements, we examined both the repeat element spanning the editing site and the total repeat elements within the edited lncRNAs (Figure 3I). Notably, over 80% of the editing sites in the PreVOC and Omicron infected patients were located within the Alu repeats, whereas only approximately 10% of the editing sites were within Alu repeats in the Delta group (Figures 3J; Tables S2A–S2C). Intriguingly, a substantial fraction of the editing sites in the Delta group were either within rRNA repeats or outside of any repeat regions, in contrast to the PreVOC or Omicron patients. The abundance of rRNA repeats was also significantly higher in the Delta infected patients compared to its distinct genomic distribution as well as other groups (Figures 3K; Table S4). Moreover, Alu and long interspersed nuclear element 1 (LINE1) repeats exhibited significantly higher abundance in all the groups compared to their genomic distribution, whereas long terminal repeats (LTRs) were consistently lower in all the groups (Figure 3K). Interestingly, the abundance of all repeats in the PreVOC group was comparable to that of the Omicron group, indicating a striking difference in repeat abundance in the lncRNAs edited in the Delta patients. This suggests that repeats may serve as modulators or decisive factors for a gene to undergo editing.

### Functional outcomes of editing in host lncRNAs across the SARS-CoV-2 variants

**Figure 4:**
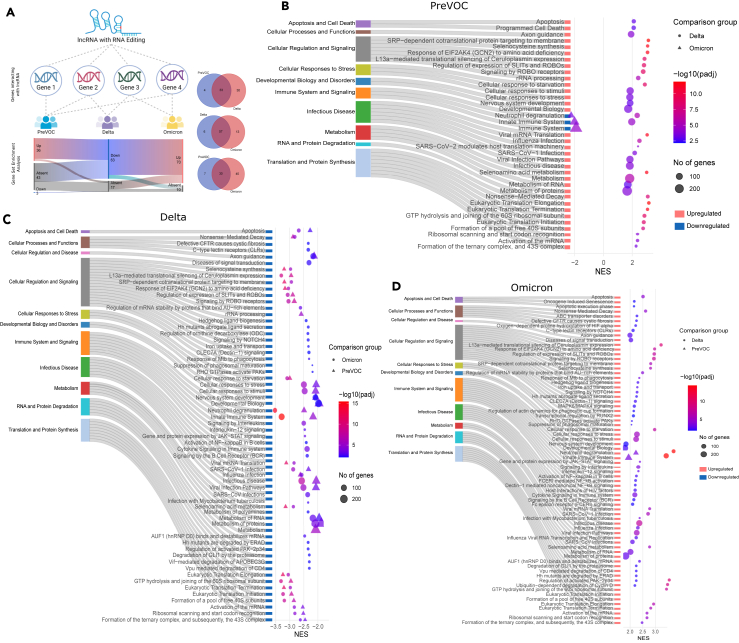
Variant specific functional effect of editing in host lncRNA (A) Analyzing functional effects of RNA editing by gene set enrichment analysis of genes interacting with the edited lncRNAs. (B–D) Biological functions perturbed by the RNA editing as identified through GSEA in (B) PreVOC, (C) Delta, and (D) Omicron groups. Biological functions grouped into functional categories are on the left and are colored individually. Normalized enrichment score (NES) is represented on the x axis and upregulation/downregulation of pathways are marked by red and blue box, respectively. The shape of individual pathways represents the comparison group. The color of the pathway represents the significance while the size represents the number of genes involved in the pathway.

Up to this point, we have identified the DE of the RNA editing enzymes and underlying distribution of repeats as potential modulators of differential RNA editing across the three studied SARS-CoV-2 variants. Yet, does this differential editing yield any functional outcomes? And if so, what are the potential mechanisms supported by genomic evidence (Figure 3L)? To elucidate the functional impact of RNA editing within the host lncRNAs, we retrieved genes interacting with the edited lncRNAs. Using the expression of these interacting genes, we conducted gene set enrichment analysis (GSEA) with the Reactome database (Figure 4A; Tables S5A–S5C). The individual pathways were then categorized into functional groups. In the PreVOC group, all pathways except three related to the immune system and signaling were upregulated compared to the Delta and Omicron groups (Figure 4B). In the Delta, all pathways were downregulated compared to the PreVOC and Omicron infected patients, while in the Omicron, all pathways were upregulated compared to the PreVOC and Delta (Figures 4C and 4D). Overall, a total of 30 pathways primarily corresponding to infectious disease, cellular signaling, metabolism, and translation processes were upregulated in the PreVOC and Omicron but downregulated in the Delta. These functional categories are closely associated with the disease severity and outcome. The shift in the functional profile of the edited lncRNAs across the PreVOC, Delta, and Omicron highlights how RNA editing can modulate disease pathophysiology.

### Genomics based mechanistic understanding of RNA editing-mediated function of lncRNA across the SARS-CoV-2 variants

**Figure 5:**
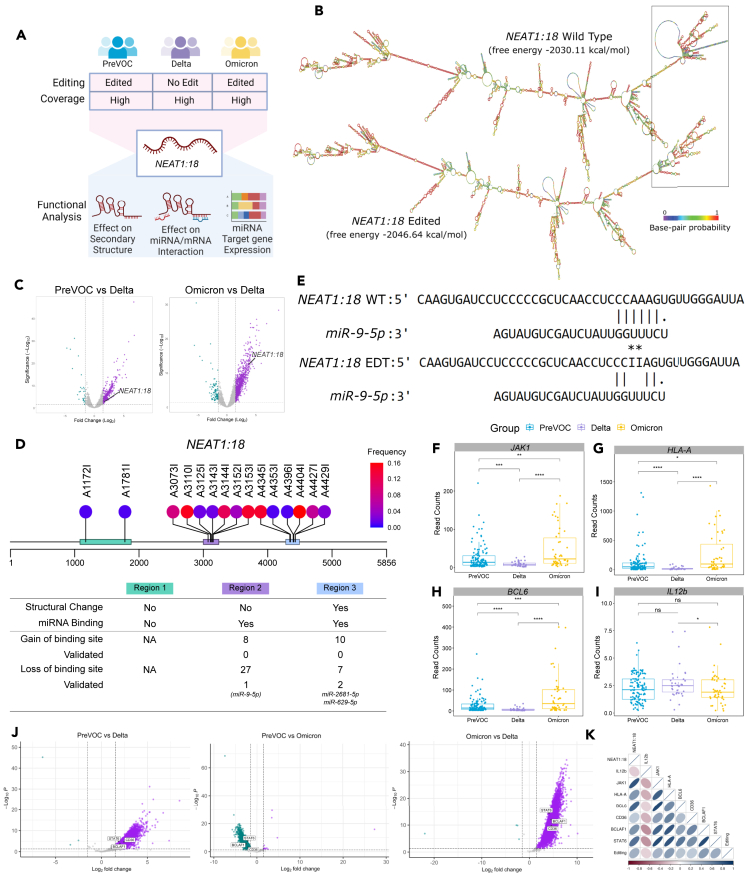
Mechanistic elucidation of the functional role of RNA editing (A) Graphical representation with selection criteria and mechanistic investigation of editing in *NEAT1:18*. (B) Secondary structure analysis of the wild-type as well as edited *NEAT1:18* transcript. The color of each base represents the base pair probability. The structural change between the wild-type and the edited transcript is highlighted in the box. (C) Volcano plot showing differential expression of *NEAT1:18* in the PreVOC-Delta and Omicron-Delta comparison, where the fold change applies to the first group. (D) Editing sites in *NEAT1:18* and its functional outcome. The dots represent the editing site, with the color of the dots representing the frequency of editing at that site, while the color of the box represents regions with a high number of RNA editing sites. (E) miRNA binding site analysis of *NEAT1:18* wild-type as well as edited transcript, showing loss of binding site for *miR-9-5p* due to two editing: A3152I and A3153I. (F–I) Normalized expression (read counts) of *miR-9-5p* target genes. (F) *JAK1*, (G) *HLA-A*, (H) *BCL6*, and (I) *IL12b* across the PreVOC, Delta, and Omicron group. The Mann-Whitney U test was applied for comparing gene expression across the groups. Significance value is denoted as ∗, where ∗ indicates *p* ≤ 0.05, ∗∗ indicates *p* ≤ 0.01, ∗∗∗ indicates *p* ≤ 0.001 and ∗∗∗∗ indicates *p* ≤ 0.0001. (J) Volcano plot showing differential expression of *CD36*, *STAT6*, and *BCLAF1* in the PreVOC-Delta and Omicron-Delta comparison, where the fold change applies to the first group. (K) Pearson correlation analysis of the *NEAT1:18* editing and expression of *NEAT1:18*, as well as genes perturbed by the editing within *NEAT1:18*.

To explore the mechanisms of RNA editing mediated change in the biological function, we selected *NEAT1:18*, a lncRNA transcript with high coverage in all the three groups and were edited in the PreVOC and Omicron but no edit in the Delta group (Figure 5A). A total of three editing regions were identified in *NEAT1:18*: the first region harbors 2 editing sites (A1172I and A1781I), the second region harbors 7 editing sites (A3073I, A3110I, A3125I, A3143I, A3144I, A3152I, and A3152I), and the third region harbors 6 editing sites (A4345I, A4353I, A4396I, A4404I, A4427I, and A4429I). We computed the RNA secondary structure of both the wild-type and the edited transcript, wherein a structural change was observed in the edited transcript at the third region compared to the wild-type one. The change in structure also resulted in a change in the free energy (ΔG −13.53 kcal/mol) in the edited transcript. The change in free energy of the edited transcript indicated that the edited transcript is more stable compared to its wild type (Figure 5B). Since increased stability of a transcript is more likely to improve the half-life of the transcript, it may persist in the cell for a longer duration before being degraded. This extended lifespan may allow the RNA to participate in the cellular processes for a more extended period, and possibly contributing to higher expression levels. Therefore, to check whether the edited lncRNAs have a different expression pattern across the SARS-CoV-2 variants, we performed DE analysis of the lncRNAs (Tables S6A–S6C). Interestingly, we observed *NEAT1:18* to be upregulated in both the PreVOC and Omicron group compared to the Delta (Figure 5C), and *NEAT1:18* expression showed a significant positive correlation with its editing (R^2^ = 0.4, *p* = 0.002), supporting the hypothesis of editing-mediated increased stability of lncRNA might lead to change in expression as well. Moreover, we assessed the intersection between the edited and differentially expressed lncRNAs to understand the impact of RNA editing on lncRNA expression. While there was limited overlap between edited and differentially expressed lncRNAs (Figures S3A–S3C), indicating that not all editing events result in significant expression changes, a substantial number of edited lncRNAs exhibited altered expression across the groups (Figures S3D and S3E). This suggests that while editing may not uniformly influence the stability of all lncRNAs to induce significant expression changes, it still contributes to noticeable alterations in the lncRNA expression levels.

Since lncRNAs are known to bind both miRNAs and mRNAs to regulate their expression, and RNA editing may impact the miRNA/mRNA binding, we investigated the potential miRNA and mRNA binding sites within the *NEAT1:18* transcript, and the possible effect of RNA editing on the lncRNA-miRNA/mRNA interaction. We first checked whether the miRNA binding site overlaps with any editing sites within the transcript and potential effect of editing on the miRNA binding. We identified a total of 34 miRNAs that lost their binding sites due to editing in the different regions of *NEAT1:18*. Out of these, 3 miRNAs are experimentally validated to bind with the native form of *NEAT1* (Figures 5D; Tables S7A–S7C). Among the 3 miRNAs, we focused on *miR-9-5p*, which lost its binding site due to A-to-I editing at the 3152 and 3153 positions of the *NEAT1:18* transcript (Figures 5D and 5E), suggesting an elevated level of free *miR-9-5p* in the PreVOC and Omicron group. To evaluate the potential effect of the higher level of *miR-9-5p*, we examined the expression of experimentally validated target genes of *miR-9-5p* across the groups. It has been shown in independent studies that miR-9-5p positively regulates, The Janus kinase (*JAK*)*1*, *HLA-A*, and *BCL6*, and negatively regulates *IL12b*. Importantly, from our own data, we found that the expressions of *JAK1*, *HLA-A*, and *BCL6* genes were upregulated in the PreVOC and Omicron group, whereas the expression of *IL12b* was downregulated in the Omicron patients compared to the Delta (Figures 5E–5H).

Next, we checked the potential mRNA interaction with *NEAT1:18*, and identified three mRNAs, *CD36*, Signal transducer of activation (*STAT*)*6*, and *BCLAF1*, to have experimentally validated interaction with *NEAT1* as reported in the NPInter database, which documents functional interactions between the ncRNAs (except tRNAs and rRNAs) and biomolecules (proteins, RNAs, and DNAs). We used the IntaRNA tool, for RNA-RNA interactions, to predict the binding of these three mRNAs with both the edited as well as wild-type *NEAT1:18* and calculated the interaction energy for each binding. Interestingly, the *CD36*, *BCLAF1*, and *STAT6* were found to have higher interaction energy with the edited isoforms of *NEAT1:18* as compared to the wild-type *NEAT1:18* isoform (Table S8) and were found to have higher expression in PreVOC and Omicron group as compared to the Delta group (Figure 5J). Furthermore, the *CD36*, *STAT6*, and *BCLAF1* also showed a significant positive correlation with *NEAT1:18* expression and *NEAT1:18* editing (Figure 5K), suggesting possible editing-mediated change in the expression of *CD36*, *STAT6*, and *BCLAF1*. The 4 miRNA target genes, *JAK1*, *BCL6*, *HLA-A*, and *IL12b* were also found to have a significant correlation with the *NEAT1:18*, with a positive correlation for *JAK1*, *BCL6*, and *HLA-A*, and a negative correlation for *IL12b* (Figure 5K). JAK1 and STAT6 are central molecules in the JAK/STAT pathway, whereas BCL6 and BCLAF1 are central molecules in the apoptosis, and HLA-A, IL12b, and CD36 are key molecules in the innate immune signaling pathways. CD36 is also involved in fatty acid metabolism pathways. The upregulation of these pathways in the PreVOC and Omicron infected patients supports the proposed hypothesis of differential RNA editing-mediated modulation of biological functions across the different variants of the pathogen, SARS-CoV-2.

## Discussion

Currently, over 170 RNA base modifications have expanded the regulatory repertoire of the four RNA nucleotides to more than a hundred functional nucleotides. While A-to-I editing is the most abundant RNA editing type in the human transcriptome, C-to-U editing is primarily observed in the viral RNA, with the most recent evidence of C-to-U editing mediated nucleotide diversity in SARS-CoV-2 virus.^51^ It has previously been shown that A-to-I RNA editing controls the fate of several RNA viruses, such as the measles virus, human immunodeficiency virus 1 (HIV-1), hepatitis C virus (HCV), and influenza A.^5^^,^^52^^,^^53^ The functional/regulatory effect can be both pro- and antiviral depending on the specific, yet dynamic host-pathogen interaction scenario. During an infection, RNA editing serves as a mechanism for the host to distinguish between the self- and viral RNA, facilitating effective sensing by intracellular sensors like RIG-1, MDA5, Toll-like receptor (TLR), and RIG-I-like receptors (RLR).^54^ This process is not limited to the virus alone; host RNAs are also edited to combat the infection. Chronic inflammatory circumstances increase RNA editing, which stabilizes proinflammatory transcripts and puts “fuel on the fire” for the ongoing inflammatory response.^55^ Despite its potential impact on disease progression, the role of RNA editing in the host during an infection needs deeper investigation in different disease contexts. Recent studies on SARS-CoV-2 infection-mediated editing of host RNA have presented conflicting findings. Crooke et al. reported reduced A-to-I editing,^7^ while Rabinowicz et al. reported *ADAR1*-mediated increased A-to-I editing in the host transcriptome during infection.^25^ Another recent study by Kurkowiak et al. investigated viral RNA editing as well as host interferon response across the SARS-CoV-2 variants of concern.^6^ *ADAR1p150* and *ADAR1p110* are two isoforms transcribed by alternate promoter usage, wherein *ADAR1p150* itself is coded by 2 different transcripts: *ADAR-202* and *ADAR-208*. Furthermore, there are 13 more protein coding transcripts of *ADAR1* and isoform-specific usage of *ADAR1*, and their role requires dedicated investigation from an alternative transcription perspective and also isoform-specific investigation at the protein level.

Our study delves into the RNA editing dynamics in the 211 hospital-admitted COVID-19 patients across the three variants of concern: PreVOC, Delta, and Omicron. While previous studies reported differential editing in the host mRNA between the infected and healthy individuals, our findings revealed no significant difference in the mRNA editing across the three virus variants. This may be due to the study involving different patient cohorts which highlights the importance of multiple studies across the globe in different populations to capture the dynamics. Additionally, there was no notable difference in editing frequency between the mRNA and lncRNA in the Delta and Omicron groups. However, a remarkable distinction in editing frequency within the lncRNAs was observed among the patients infected with the three SARS-CoV-2 variants. This observation gains significance as the majority of editing sites are within the repeats, particularly Alu, and lncRNAs generally contain more repeats than the mRNAs.

Importantly, we did not find any relation between the editing frequency and the clinical parameters, or vice versa in any of the patient groups, despite some of the clinical parameters being significantly different across the groups. This is possibly because the samples used in this study were collected from the patients when they reported to the hospital for the first time after the onset of symptoms. The clinical parameters were also recorded at the time of hospital admission. RNA editing is a dynamic host response, which is a part of initial host defense mechanism, set within days or even hours post infection.^25^ Through our analysis, we have shown that the edited lncRNAs have potential to regulate different biological processes important in the infectious disease context. However, editing-mediated perturbation of biological processes, and translation of the specific perturbation into observable clinical phenotype may not be possible at the very early stage of infection/time of hospital admission, and require at least one more data point during the disease progression phase. Consequently, it suggests that lncRNA editing is more closely associated with the pathogen variant rather than the clinical phenotypes and may contribute to differences in the observed disease severity.

We observed a distinctly different RNA editing pattern in the Delta group, characterized by differences in the overall editing frequency, types of RNA editing, and the number of edited lncRNAs compared to the PreVOC and Omicron infected patients. Notably, the Delta patients exhibited a reduced interferon response and an overall sub-optimal immune reaction compared to the PreVOC and Omicron patients. While the expression of editing enzymes correlated directly with editing in the PreVOC and Omicron groups, the Delta patients displayed a dissimilar pattern. The Delta group also stood out in terms of the presence of repeat elements within the edited lncRNAs, with the majority of editing sites located outside of the repeat elements, as opposed to the PreVOC and the Omicron patients. This peculiar RNA editing pattern in the Delta patients may be attributed to the distinctive host response elicited by the SARS-CoV-2 Delta variant, wherein the immune response is suppressed, leading to a reduced or delayed humoral immune response and increased disease severity.^56^^,^^57^ Another possible explanation to this is that the observed editing in the Delta patients is possibly resulting from the off-target activities of the editing enzymes, especially since we observed a high abundance of C-to-U editing (C>U and G>A), which are reported as “non-adaptive” RNA editing. The overall reduced RNA editing in the Delta patients also aligns with the findings from Crooke et al., who demonstrated suppressed RNA editing in the severe COVID-19 infections.^58^ Additionally, a switch in the functional profiles of the edited lncRNAs, especially key pathways such as infectious disease, cellular signaling, metabolism, and translation processes, which are essential in infectious disease, suggest RNA editing as an important factor in modulating disease severities.

It is recognized that lncRNAs can lead to the dysregulation of critical target genes by engaging in specific interactions at different hierarchies of DNA, RNA (mRNA/miRNA), and protein. Editing in RNA transcripts may also manifest change in the folding of RNA secondary structures, influencing their structural stability and potential accessibility for subsequent processing. On the other hand, some of the editing may not have any functional outcome at all.^2^ This is evident as we observe a site-specific editing-mediated structural change and stability as well as loss of miRNA binding sites on the *NEAT1:18* transcript (region 2 and region 3), while editing in yet another region (region 1) did not have any functional outcomes. The editing-mediated structural change may alter the stability of the edited transcript, which in turn, may lead to changes in the expression of the particular transcript. It was observed that genes with the more stable RNAs have increased expression levels even after transcription inhibition, suggesting that the increased expression of *NEAT1:18* in the PreVOC and Omicron group could be due to the A-to-I editing within the transcript. However, further validation is required to understand the relationship between the stability and expression of specific transcripts. *NEAT1* is reported to increase the innate immune response, thereby increased *NEAT1:18* suggests an increased immune response in both the PreVOC and Omicron, but not in the Delta group, serving as a classic example of fuel-on-fire, where initial increased immune and interferon response led to increased expression of *ADAR1*, and subsequently increased RNA editing, which in turn, lead to increased expression of immune-modulatory transcripts. Therefore, it is imperative to look into the lncRNA-specific functional importance of RNA editing events, especially since both *NEAT1* and *UGDH-AS1*, the two lncRNAs with high editing frequency, are reported to be involved in multiple infectious diseases. However, the function of the transcripts with the highest number of editing in each group (*lnc-BICRAL-3:1* in the PreVOC, *lnc-FRG1-8:1* in the Delta, and *lnc-NPIPB12-1:1* in the Omicron) are yet to be determined. Our study provides genomics-based evidence toward a differential host RNA editing in response to different variants of the same pathogen, SARS-CoV-2. Importantly, our study highlights the role of lncRNAs and how editing within the lncRNAs modulates the COVID-19 disease severity. Furthermore, our study suggests that different RNA editing within the same transcript may have different functional consequences and therefore, site-specific functional impact of RNA editing should be investigated more closely in future studies.

### Limitation of the study

Specific investigations including gain/loss of miRNA binding sites and subsequent effect on the miRNA target expression, as well as 3′ and 5′ UTR mediated lncRNA interaction studies could shed light on the function of these lncRNAs. Unfortunately, these aspects could not be thoroughly investigated in the current study due to the unavailability of miRNA expression data from the same dataset and the absence of reads uniformly mapping to the UTR regions. Another limitation of the study is the unavailability of the whole genome sequencing data of the same individuals, which otherwise could have provided information on whether a particular RNA editing event is an attempt to create diversities within the transcript, or restoration of an existing SNP back to its original form. Furthermore, the number of lncRNAs edited in the Delta group is small compared to the PreVOC and Omicron infected group, which may have an impact on the overall proportion of different editing types and abundance of repeat elements. These limitations should be acknowledged in the interpretation of the study results.

## STAR★Methods

### Key resources table

**Table:** 

REAGENT or RESOURCE	SOURCE	IDENTIFIER
QIAmp viral mini kit	Qiagen	Cat# 52906
TRUPCR® SARS-CoV-2 RT qPCR Kit	3B BlackBio	Cat# 3B304
Illumina TruSeq® Stranded Total RNA Library Prep Gold	Illumina	Cat# 20020599
AMPure XP	Beckman Coulter	Cat# A63881
Agilent 2100 Bioanalyzer	Agilent	Cat# 5067-4626
Qubit dsDNA HS Assay kit	Invitrogen	Cat# Q32854
NextSeq 2000 P2 sequencing reagent kit	Illumina	Cat# 20046813

**Deposited data**		

Raw bulk RNAseq data	This paper	SRA: PRJNA678831, PRJNA868733 and PRJNA952815

**Software and algorithms**		

bcl2fastq 2.19	NA	GitHub - brwnj/bcl2fastq: NextSeq specific bcl2fastq2 wrapper.
Trimmomatic v0.39	Bolger et al.^59^	https://github.com/usadellab/Trimmomatic
Burrows-Wheeler Aligner	Li et al.,^60^	https://github.com/lh3/bwa
SPRINT	Zhang et al.,^48^	https://github.com/jumphone/SPRINT
Salmon	Patro et al.,^61^	https://github.com/COMBINE-lab/salmon
DESeq2	Love et al.,^62^	https://github.com/thelovelab/DESeq2
NextClade	Aksamentov et al.,^63^	https://github.com/nextstrain/nextclade
RepeatMasker	Tarailo-Graovac and Chen et al.,^64^	https://www.repeatmasker.org/cgi-bin/WEBRepeatMasker
Dfam 3.0	Storer et al.,^65^	https://www.dfam.org/
NPInter v5.0	Zheng et al.,^66^	http://bigdata.ibp.ac.cn/npinter5
fgsea		https://github.com/ctlab/fgsea
RNAfold	Gruber et al.,^67^	http://rna.tbi.univie.ac.at/cgi-bin/RNAWebSuite/RNAfold.cgi
lncRNASNP v3	Yang et al.,^68^	http://gong_lab.hzau.edu.cn/lncRNASNP3/#!/
miRNet 2.0	Chang et al.,^69^	https://www.mirnet.ca/
DIANA-LncBase v3	Karagkouni et al.,^70^	https://diana.e-ce.uth.gr/lncbasev3/home
IntaRNA	Mann et al.,^71^	https://rna.informatik.uni-freiburg.de/IntaRNA/Input.jsp
Prism 9	GraphPad	https://www.graphpad.com/
R 4.2	CRAN	https://www.r-project.org/
SRplot	Tang et al.,^72^	http://www.bioinformatics.com.cn/srplot

### Resource availability

#### Lead contact

Further information and requests for resources and reagents should be directed to and will be fulfilled by the lead contact, Rajesh Pandey (rajesh.p@igib.res.in).

#### Materials availability

This study did not generate new unique reagents and material.

#### Data and code availability

• Raw RNAseq data have been deposited at NCBI SRA repository and are publicly available. Accession numbers are listed in the key resources table. All the data reported in this paper will be shared by the lead contact upon request.
• This paper does not report any original code.
• Any additional information required to reanalyze the data reported in this paper is available from the lead contact upon request.

### Experimental model and study participant details

#### Human subjects and clinical protocol

Patients admitted to a tertiary care center (Max Super Speciality Hospital, Delhi, India) during different time periods of COVID-19 pandemic (April 2020 to March 2022) were retrospectively enrolled for the study. The age and gender distribution are available in Table S1. In this study, the median age was 58 years for the PreVOC group, 64 years for the Delta group and 38 years for the Omicron group. The gender (M/F ratio) among the three groups was comparable. The samples were anonymized, and the comprehensive clinical and demographic data were recorded electronically. Institutional ethical clearance for the study was obtained from both CSIR-IGIB and the Max Super Speciality Hospital. The studies involving human participants were reviewed and approved by CSIR-IGIB’s Human Ethics Committee Clearance (Ref No: CSIR-IGIB/IHEC/2020-21/01). The study was conducted following the guidelines of the Declaration of Helsinki. The patients/participants provided their written informed consent to participate in this study.

#### Collection and classification of clinical samples

Nasopharyngeal swabs were collected in Viral Transport Media (VTM) upon admission, followed by the RT-PCR for SARS-CoV-2. Viral RNA from VTM was isolated using the QIAmp viral mini kit (Qiagen, Cat. No. 52906) and SARS-CoV-2 detection and quantification was performed using TRUPCR SARS-CoV-2 kit (3B BlackBio Biotech India Ltd., Cat. No. 3B304), with a cycle threshold of 35. The SARS-CoV-2 genome sequencing was performed for these RT-PCR positive samples using Oxford Nanopore Mk1c and Illumina NextSeq 2000 for identification of the virus variants. The clinical and demographic data were collected from the electronic health record (EHR) as per standard practice. Based on the clinical data and the infection status, a total of 211 patents were identified, and grouped into 3 patient groups based on the variants detected: PreVOC (n = 125), Delta (n = 39) and Omicron (n = 47).

### Method details

#### Library preparation and sequencing

RNA sequencing libraries were prepared with Illumina TruSeq® Stranded Total RNA Library Prep Gold (cat. no 20020599) using a total of 250 ng RNA isolated from the nasopharyngeal swabs of the COVID-19 patients, as per manufacturer’s reference guide (1000000040499 v00). Briefly, cytoplasmic and mitochondrial rRNA was removed using the Illumina Ribo-Zero plus rRNA Depletion Kit, followed by the RNA fragmentation, first strand cDNA synthesis, RNA strand digestion and synthesis of the second strand cDNA. The blunt 3’ end of the double stranded cDNA was then repaired and polyadenylated prior to the index adapter ligation. The library was purified/size selected using AMPure XP (Beckman Coulter, A63881), followed by the quality check for appropriate fragment size (∼280 base pairs) using Agilent 2100 bioanalyzer and High Sensitivity DNA Kit and quantity check using the Qubit dsDNA high sensitivity assay kit. The libraries were sequenced on NextSeq 2000 platform using the P2 sequencing reagent kit, with 2 x 151 cycles of sequence by synthesis (SBS) and at a final loading concentration of 650 pM.

#### Quality control, mapping to reference and identification of RNA editing events

The initial quality assessment of raw sequencing reads was conducted using FastQC, and subsequent removal of adapter sequences was performed using Trimmomatic.^59^ Prior to identifying editing sites, to ensure stringent read quality, we first performed read filtering, removing reads with a phred score < 30 as well as poor quality bases were trimmed. Therefore, all the reads selected for the analysis had a minimum error rate of 0.001 per base. Next, we used SPRINT to align the reads against the reference (GRCh38.p14 and LNCipedia 5.2 hc) and identify the editing site. SPRINT uses bwa as the aligner, and only uniquely mapped reads with zero gaps and a minimum mapping score of 25 were selected for the identification of RDD events. Editing frequency was determined by normalizing the number of edited bases at a specific position with the total bases at that position. To eliminate potential false positives, RNA editing events with fewer than 3 reads and a frequency below 0.25 were excluded.

#### Differential gene expression analysis

Trimmed sequencing reads were taken for differential gene expression analysis. The reads were aligned to the human genome using the Salmon quasi-mapping tool to quantify read abundance or transcript expression levels. DESeq2 was employed for the differential gene expression analysis, utilizing Wald's statistics test. Genes with a *p*-adjusted value of ≤ 0.05 and a Log2 fold change of ≥ ± 1.5 were considered differentially expressed.^62^

#### Viral mutation analysis

High quality SARS-CoV-2 genome sequences (>80% coverage) from the enrolled patients were used for viral mutation analysis. Mutation in the viral genome was identified using NextClade taking the Wuhan-Hu-1/2019 genome (MN908947) as the reference. Frequencies of the single nucleotide variants (SNVs) were calculated by normalizing the number of times a particular SNV occurred with the total number of SNVs in that group. The bases adjacent to the SNV sites were fetched from the Wuhan-Hu-1/2019 genome sequence.

#### Repeat element enrichment analysis

Repeat elements within the edited lncRNAs were identified using the RepeatMasker web server.^64^ The analysis was performed using rmblast algorithm and Dfam 3.0 database.^73^ The repeat elements were segregated with respect to class and subclass, and abundance was represented as % of the total repeats. Both, repeats spanning the editing sites and total repeats within the edited lncRNAs, were calculated and compared against the overall repeat abundance within the noncoding human genome.

#### Gene set enrichment analysis

Genes interacting with the edited lncRNAs were fetched from the experimentally validated database of lncRNA interaction: NPInter.^74^ Gene set enrichment analysis (GSEA) was performed on the interacting genes using the fgsea package taking into account the expression of the genes.^75^ ReactomeDB was used to fetch the biological pathways associated with the gene sets.

#### RNA structure analysis

RNA secondary structure and minimum free energy was calculated using the RNAfold web server.^67^ Isolated base pairing was excluded while possible G-quadruplex structures were included in the structure prediction algorithm for calculation of the minimum free energy.

#### miRNA and mRNA binding site analysis

The impact of RNA editing within the lncRNA on miRNA binding was evaluated using the lncRNASNP v3 database.^68^ Sequences of the lncRNAs, both with and without RNA editing, were used to identify the gain and loss of the miRNA binding sites. Subsequently, miRNAs associated with alterations in binding due to editing were cross verified for experimentally validated lncRNA interactions using miRNet and DIANA Tools.^69^^,^^76^ Experimentally confirmed miRNAs exhibiting changes in binding sites were further investigated for their mRNA targets using miRNet.

To assess the impact of RNA editing on mRNA binding to the edited lncRNA, we first identified the mRNAs having experimentally validated binding sites on *NEAT1*. Two stretches, each of 200 bps covering the editing sites on *NEAT1:18* transcripts, were used to predict the binding sites and interaction energy using the IntaRNA tool.^71^

### Quantification and statistical analysis

The data was described using descriptive statistics, which display continuous variables as medians or interquartile ranges and categorical variables as percentages or proportions. Wherever appropriate, we compared the differences using the Mann–Whitney U test and Chi-square testing. We used the Mann–Whitney U test to compare some of the clinical parameters (Age, SpO_2_, Length of hospital stay, CRP), editing frequency and expression of specific genes across the groups. The significance for proportion data, including the gender distribution, shortness of breath, patient requiring respiratory support, repeat element abundance, were calculated using Chi-square test. For linear regression analysis, ANOVA was used to test the significance. The Mann–Whitney U test and Chi-square tests were performed using a licensed version of GraphPad Prism 9.0. The linear regression analysis and ANOVA were performed using the base R. SRplot was used for the Pearson correlation analysis and visualization of the data. Significance value is denoted as ∗, where ∗ indicates *p* ≤ 0.05, ∗∗ indicates *p* ≤ 0.01, ∗∗∗ indicates *p* ≤ 0.001 and ∗∗∗∗ indicates *p* ≤ 0.0001.

## Supplemental information

Document S1. Figures S1–S3

Table S1. Clinical Information

Table S2. RNA Editing in lncRNAs and mRNAs across VOCs

Table S3. List of viral SNVs

Table S4. Repeats within edited lncRNA

Table S5. GSEA of the edited lncRNAs in three variants

Table S6. DE analysis of lncRNAs

Table S7. Binding site analysis

Document S2. Table S8. mRNA interaction sites on NEAT1:18, Related to Figure 5
