# Supplementary material for: RNA editing in host lncRNAs as potential modulator in SARS-CoV-2 variants-host immune response dynamics
Source: iScience. 2024 Apr 29;27(6):109846. doi: 10.1016/j.isci.2024.109846 (PMC11103575; doi:10.1016/j.isci.2024.109846)
Supplement: Document S1. Figures S1–S3 [file mmc1.pdf]

iScience, Volume *27*

Supplemental information

RNA editing in host lncRNAs as potential modulator in SARS-CoV-2 variants-host immune response dynamics

Partha Chattopadhyay, Priyanka Mehta, Kanika, Pallavi Mishra, Chinky Shiu Chen Liu, Bansidhar Tarai, Sandeep Budhiraja, and Rajesh Pandey

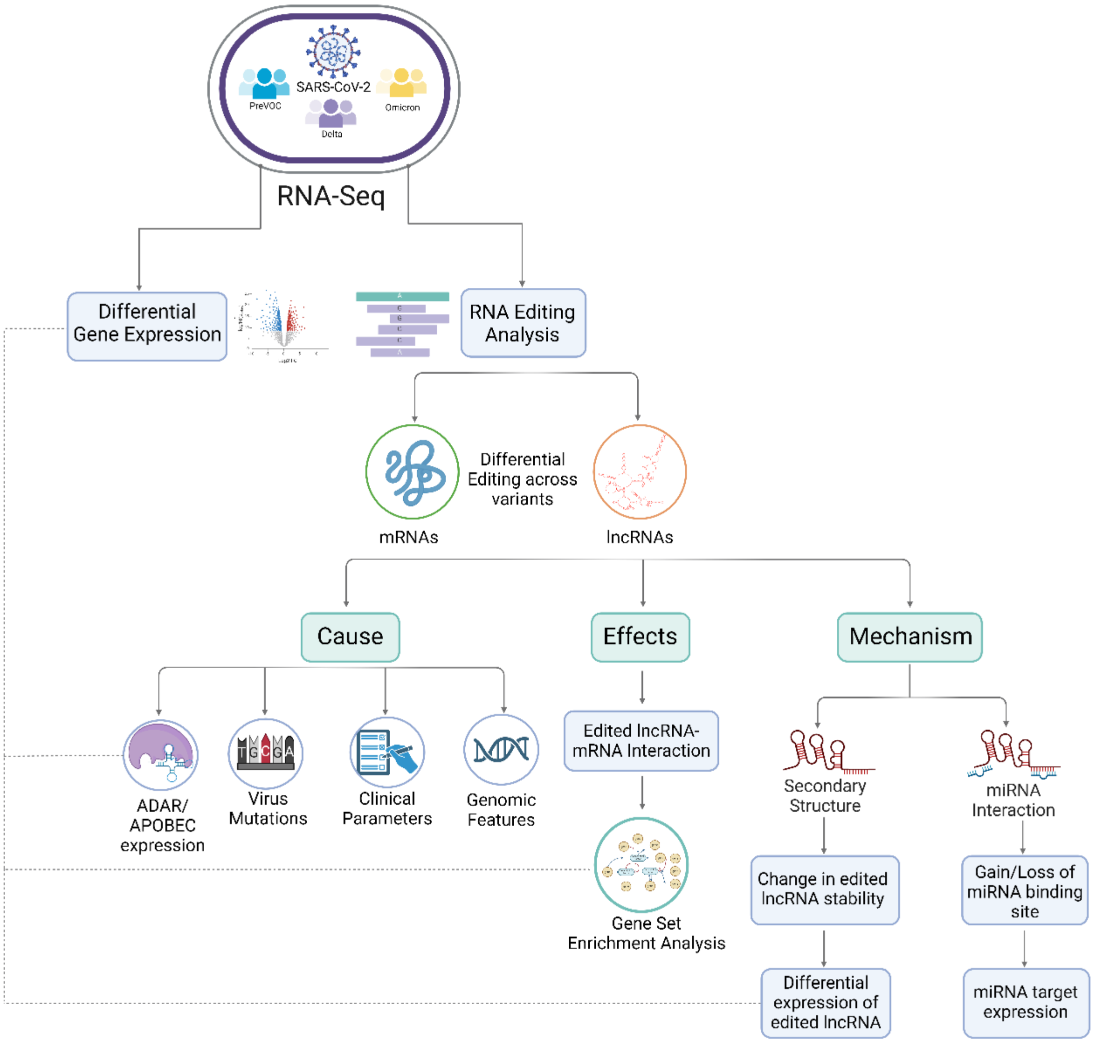

Supplementary Figure S1: Graphical representation of the experimental methodologies and analytical pipeline for identification of differential RNA editing across SARS-CoV-2 variants. Related to STAR Methods

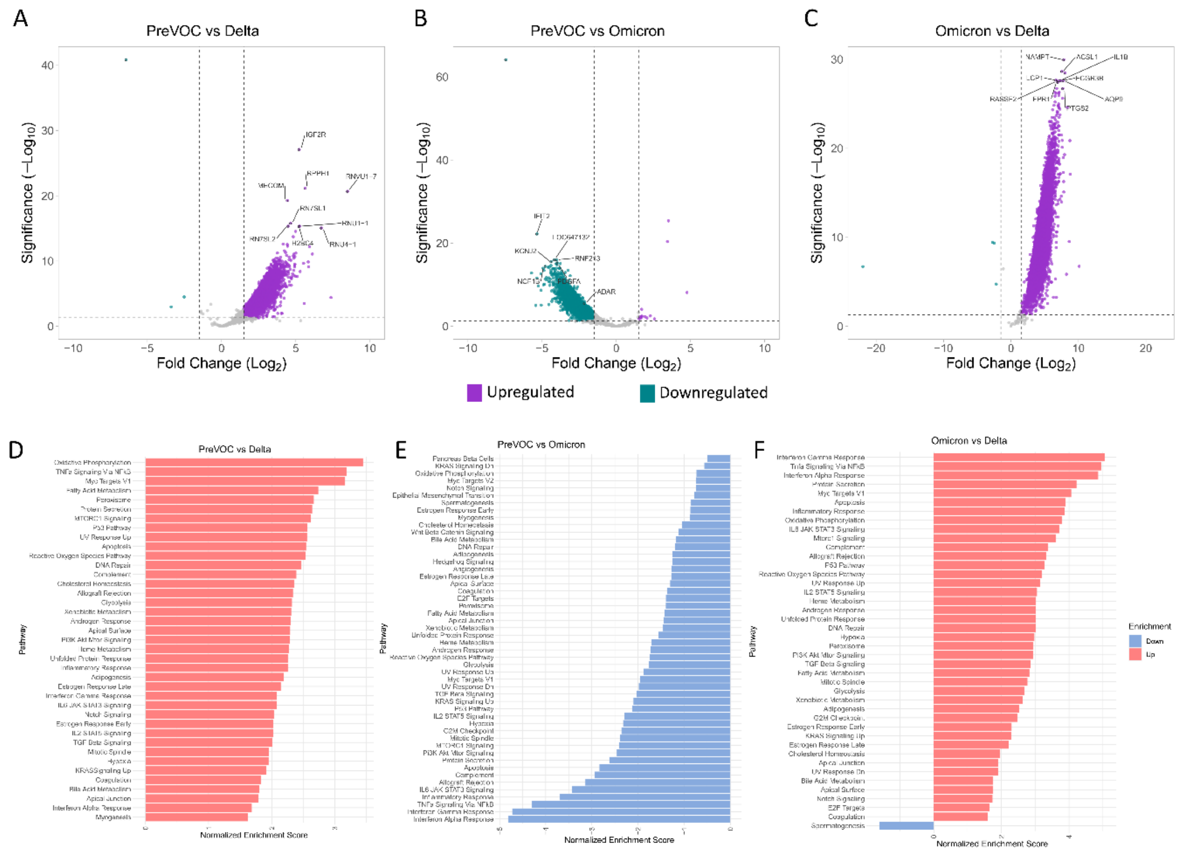

Supplementary Figure S2: Differential expression and gene set enrichment analysis of genes across PreVOC, Delta and Omicron. Related to Figure 2

(A-C) Volcano plot showing differential expression of genes in (A) PreVOC vs Delta, (B) PreVOC vs Omicron, and (C) Omicron vs Delta comparison group (fold change applies to the first group in comparison). (D-F) GSEA of genes differentially expressed between (E) PreVOC vs Delta, (F) PreVOC vs Omicron, and (G) Omicron vs Delta.

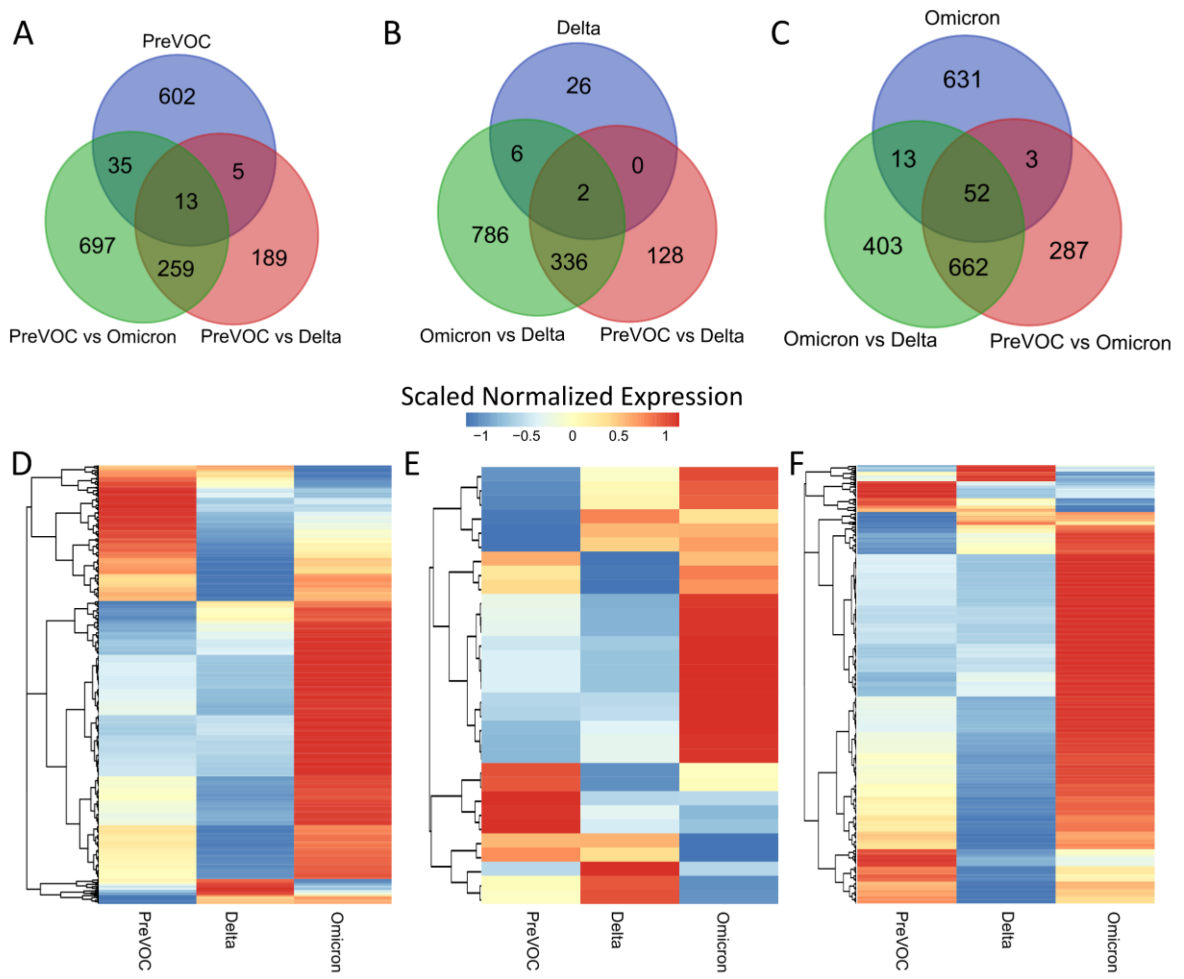

Supplementary Figure S3: Expression profile of edited lncRNAs across SARS-CoV-2 variants. Related to Figure 2

(A) Overlap of lncRNAs edited in PreVOC group with the lncRNAs differentially expressed between PreVOC/Delta and PreVOC/Omicron comparison. (B) Overlap of lncRNAs edited in Delta group with the lncRNAs differentially expressed between Omicron/Delta and PreVOC/Delta comparison. (C) Overlap of lncRNAs edited in Omicron group with the lncRNAs differentially expressed between Omicron/Delta and PreVOC/Omicron comparison. (D-F) Overall expression of lncRNAs edited in the (D) PreVOC, (E) Delta and (F) Omicron group. Expression is represented as scaled averaged normalized read counts.

List of Supplementary Figures and Tables

Supplementary Figure 1: Experimental Methodology

Figure 2A-2C: Volcano plot of DEGs

Figure 2D-2F: GSEA of DEGs

Supplementary Figure 3A-3C: Volcano plot of differentially expressed lncRNAs

Supplementary Figure 3D-3F: Heatmap of expression of lncRNAs edited in each groups

Supplementary Table S1: Clinical Information

Supplementary Table S2A: RNA editing in lncRNA in PreVOC group

Supplementary Table S2B: RNA editing in lncRNA in Delta group

Supplementary Table S2C: RNA editing in lncRNA in Omicron group

Supplementary Table S2D: RNA editing in lncRNA in PreVOC group

Supplementary Table S2E: RNA editing in lncRNA in Delta group

Supplementary Table S2F: RNA editing in mRNA in Omicron group

Supplementary Table S3: List of viral SNVs

Supplementary Table S4: Repeats within edited lncRNA

Supplementary Table S5A-S5C: GSEA of the edited lncRNAs in three variants

Supplementary Table S6A-S6C: DE analysis of lncRNAs

Supplementary Table S7A-S7C: Binding site analysis
