# Supplementary material for: RNA editing in host lncRNAs as potential modulator in SARS-CoV-2 variants-host immune response dynamics
Source: iScience. 2024 Apr 29;27(6):109846. doi: 10.1016/j.isci.2024.109846 (PMC11103575; doi:10.1016/j.isci.2024.109846)
Supplement: Document S2. Table S8. mRNA interaction sites on NEAT1:18, Related to Figure 5 [file mmc9.pdf]

Supplementary Table S8: mRNA interaction sites on NEAT1:18. Related to Figure 5

```
STAT6
          9698                                                                                                                                                  9843
             |                                                                                                                                                     |
5'-GGG...AAAA    CA                                               A   AUA         C   GU                 C   C       C  A     UC          A    A      CC      GGUC...AAA-3'
             AAGGC  GGCAUGGUGGUUCAUGCCUGUAAUCCCAGCACUUUGGGAGGCCGAG CGG     GAUCAC UGAG  CAGGAGUUCGAGACCAG CUGG CAACAUGG AA ACCCCG  UCUACUAAAA CAAA AAAUAG  AGGAGU
             |||||  ||||::||||:|||::|||||||||||:||||::||||||||||| |||     |||||| ||||  |||:|||||||||||||| |:|| ||||||:| || |||||:  |||||||||| |||| |||:|:  |::|||
             UUCCG  CCGUGUCACCGAGUGUGGACAUUAGGGUUGUGAGGCCCUCCGGCUC GCC     CUAGUG ACUC  GUCUUCAAGCUCUGGUC GGCC GUUGUGUC UU UGGGGU  AGAUGAUUUU GUUU UUUGUU  UUUUCA
    3'-CAAAAG     AC                                                  CCCUC      A    AG                 A    C       U  C     GU                G      UA      AAAG...ACA-5'
             |                                                                                                                                                     |
            194                                                                                                                                                   49
NEAT1:18:Mut:3000-3200

interaction energy = -110.295 kcal/mol

STAT6
          7901                                                                                                                                     8029
             |                                                                                                                                        |
5'-GGG...AAUU   C                                                CCAA      C               GU     AAUC           U   U       U  A                  AU      AAAU...AAA-3'
             GGC GGGCAUGGUGGCUCACGCCUGUAAUCCCAGCACUUUGGGAGG    GGCGGG   GGAUCA  UGAG  CAGGA     GAGACCA UCC GGC AACACGG GA ACCUCGUAUCUACUAAAA   ACAAA
             ||| |||||::|||||||||:||||||||||||:|||||||||||    :|||||   ||||||  ||||  |||:|     ||||||| ||| ||| ||||||:| :| |||:|::|||||||||||   |||||
             CCG CCCGUGUCACCGAGUGUGGACAUUAGGGUUGUGAAACCCUCC    UCGCCC   CCUAGU  ACUC  GUCUU     CUCUGGU AGG CCG UUGUGUC UU UGGGGUGUAGAUGAUUUU   UGUUU
  3'-CAAAAGUU   A                                                AAC       CCU      GA    AG     CAAA       C   C           U  C                 GUU      AUUU...ACA-5'
             |                                                                                                                                        |
            192                                                                                                                                      60
NEAT1:18:WT:3000-3200

interaction energy = -97.1089 kcal/mol

BCLAF1
         25342                                                                                                                                    25471
             |                                                                                                                                       |
5'-AGA...UAAC    CA                                              ACA   U            GU         A   A   C    C      GUU  A      UC          A       AAUU...CUC-3'
             AGGC  GGCACGGUGGCUCACACCUGUAAUCCCAGCACUUUGGGAGGCUGAG    GG GGAUCA  UGAG  CAGGAGUUC AGA CAG CUGG CAACAU   GA ACCCUG  UCUAC AAAA AAACGAA
             ||||  |||||:||||||||||||||||||||||:||||::||||||||:|||    || ||||||  ||||  |||:||||| ||| ||| |:|| |||||:   :| ||||::  ||||| |||| ||||:||
             UCCG  CCGUGUCACCGAGUGUGGACAUUAGGGUUGUGAGGCCCUCCGGCUC    CC CCUAGU  ACUC  GUCUUCAAG UCU GUC GGCC GUUGUG   UU UGGGGU  AGAUG UUUU UUUGUUU
   3'-CAAAAGU    AC                                              GCCC  U      GA    AG         C   G   A    C      UCU  C      GU     A    G       GUUU...ACA-5'
             |                                                                                                                                       |
            193                                                                                                                                     60
NEAT1:18:Mut:3000-3200

interaction energy = -88.8666 kcal/mol

STAT6
          2287                                                                                                                                                      2436
             |                                                                                                                                                         |
5'-GGG...ACAG   CU        C   CU    AUUUU         U                              A      A     C    U                      C                 C         A     A        GCGC...AAA-3'
             GCG  CGCCACCA GCC  GCUA     GUUUUUGUA UUUUAGUAGAGAUGGGGUUUCACUGUGUU GCCAGG UGGU UUGA CUCCUGACCUCGUGAUCUGCC GCCUCGGCCUCCCAAAG GCUGGGAUU CAGGC UGAGCCACU
             |||  |||||||| |||  ||||     :|||||||| ||||||||||||||||||:||||::|:|| |||||| |||| |:|| |||||||||||||||||::|| |||||:||||||||||| |||::|||| ||||| :|||||||:
             CGC  GCGGUGGU CGG  CGAU     UAAAAACAU AAAAUCAUCUCUACCCCAGAGUGGUAUAA CGGUCC ACCA AGCU GAGGACUGGAGCACUAGGUGG CGGAGUCGGAGGGUUUC CGAUUCUAA GUCCG GCUCGGUGG
3'-CUC...UGUC   AC        A   AC                C                              C      G     U    U                      A                 A               C        UAUG...ACC-5'
             |                                                                                                                                                         |
            182                                                                                                                                                       39
NEAT1:18:Mut:4300-4500

interaction energy = -131.961 kcal/mol

STAT6
          2287                                                                                                                                                      2436
             |                                                                                                                                                         |
5'-GGG...ACAG   CU        C   CU    AUUUU         U                   C        A      A     C    U                      C                 C         A     A        GCGC...AAA-3'
             GCG  CGCCACCA GCC  GCUA     GUUUUUGUA UUUUAGUAGAGAUGGGGUUU ACUGUGUU GCCAGG UGGU UUGA CUCCUGACCUCGUGAUCUGCC GCCUCGGCCUCCCAAAG GCUGGGAUU CAGGC UGAGCCACU
             |||  |||||||| |||  ||||     :|||||||| |||||||||||||||||||| ||::|:|| |||||| |||| |||| |||||||||||||||||::|| |||||:||||||||||| |||::|||| ||||| ||||||||:
             CGC  GCGGUGGU CGG  CGAU     UAAAAACAU AAAAUCAUCUCUACCCCAAA UGGUAUAA CGGUCC ACCA AACU GAGGACUGGAGCACUAGGUGG CGGAGUCGGAGGGUUUC CGAUUCUAA GUCCG ACUCGGUGG
3'-CUC...UGUC   AC        A   AC                C                   A        C      G     U    U                      A                 A               C        UAUG...ACC-5'
             |                                                                                                                                                         |
            182                                                                                                                                                       39
NEAT1:18:WT:4300-4500

interaction energy = -130.847 kcal/mol

BCLAF1
         25342                                                                                                                                    25471
             |                                                                                                                                       |
5'-AGA...UAAC    CA                                          C    ACA   U            GU        CA   A    C    C      GUU  A      UC          A       AAUU...CUC-3'
             AGGC  GGCACGGUGGCUCACACCUGUAAUCCCAGCACUUUGGGAGG UGAG    GG GGAUCA  UGAG  CAGGAGUU  AGA CAG CUGG CAACAU   GA ACCCUG  UCUAC AAAA AAACGAA
             ||||  |||||:||||||||||||||||||||||:||||||||||| ||||    || ||||||  ||||  |||:||||  ||| ||| |:|| |||||:   :| ||||::  ||||| |||| ||||:||
             UCCG  CCGUGUCACCGAGUGUGGACAUUAGGGUUGUGAAACCCUCC ACUC    CC CCUAGU  ACUC  GUCUUCAA  UCU GUC GGCC GUUGUG   UU UGGGGU  AGAUG UUUU UUUGUUU
   3'-CAAAAGU    AC                                          A    GCCC  U      GA    AG        AC   G    A    C      UCU  C      GU     A    G       AUUU...ACA-5'
             |                                                                                                                                       |
            193                                                                                                                                     60
NEAT1:18:WT:3000-3200

interaction energy = -81.6018 kcal/mol

CD36
         49417                                                                                                                                        49550
             |                                                                                                                                           |
5'-AGA...UAUA  U                        A                          A    U              C                  C             C  A        C            U      AAAA...AAA-3'
             CA GGCUGGGCAUGGUGGCUCAUGCCU UAAUCCUAGUACUUCGGGAGGCUGAG CGGG   GGAUCACUUGAG UCAGGAGUUUGAGACCAG CUGGGCAACAUGG AA ACCCCAU UCUAC  GAAUAA ACAAA
             || |||||||||::|||||||||::||| ||||||:|::|||:|||||||||:||| ||||   |||||||||||| :|||:||||:|||||||| |:||||||||::| || ||||||: |||||  :||:|| |||||
             GU CCGACCCGUGUCACCGAGUGUGGA AUUAGGGUUGUGAGGCCCUCCGGCUC GCCC   CCUAGUGAACUC GGUCUUCAAGCUCUGGUC GGCCCGUUGUGUC UU UGGGGUG AGAUG  UUUGUU UGUUU
     3'-CAAAA  U                        C                          CCU            A                  A             U  C        U     AU             GUUU...ACA-5'
             |                                                                                                                                           |
            195                                                                                                                                         60
NEAT1:18:Mut:3000-3200

interaction energy = -127.1 kcal/mol

BCLAF1
          3897                                                                                                                                                 4045
             |                                                                                                                                                    |
5'-AGA...GGCU         U            C                            C  A       A     AG        CA                        A                 UC  G C        U G      AGCU...CUC-3'
             GAUUUUUGUA UUUUAGUAGAGA GGGGUUUCACCAUGUUGGCCAGGCUGGU UC AACUCCU ACCUC  GAUCCGCU  CCUCGGCCUCCCAAAGUGCUGGGAUU CAGGCGUGAGCCACCAU  CU G CUGAUAAA U UUUUCU
             :||||||||| |||||||||||| |||||:|||||||:||||||||||||| || ||||||| |||||  ||||||:|:  ||||:||||||||||||||||::||||| ||||||:||||||||||  || | |||||||| | ||||||
             UUAAAAACAU AAAAUCAUCUCU CCCCAGAGUGGUAUAACCGGUCCGACCA AG UUGAGGA UGGAG  CUAGGUGG  GGAGUCGGAGGGUUUCACGAUUCUAA GUCCGCGCUCGGUGGUA  GA C GACUAUUU A AAAAGA
3'-CUC...CCGA         C            A                            U  C       C     CA        AC                                          U   G A        C G      AUUU...ACC-5'
             |                                                                                                                                                    |
            160                                                                                                                                                  14
NEAT1:18:Mut:4300-4500

interaction energy = -141.438 kcal/mol

BCLAF1
          3897                                                                                                                                                     4045
             |                                                                                                                                                        |
5'-AGA...GGCU         U            C       C                     CUCA        A     AG        CA                        A                 UC  G C        U G      AGCU...CUC-3'
             GAUUUUUGUA UUUUAGUAGAGA GGGGUUU ACCAUGUUGGCCAGGCUGGU    AACUCCU ACCUC  GAUCCGCU  CCUCGGCCUCCCAAAGUGCUGGGAUU CAGGCGUGAGCCACCAU  CU G CUGAUAAA U UUUUCU
             :||||||||| |||||||||||| ||||||| |||||:|||||||||||||    ||||||| |||||  ||||||:|:  ||||:||||||||||||||||::||||| |||||||||||||||||  || | |||||||| | ||||||
             UUAAAAACAU AAAAUCAUCUCU CCCCAAA UGGUAUAACCGGUCCGACCA    UUGAGGA UGGAG  CUAGGUGG  GGAGUCGGAGGGUUUCACGAUUCUAA GUCCGCACUCGGUGGUA  GA C GACUAUUU A AAAAGA
3'-CUC...CCGA         C            A       A                     UAAC        C     CA        AC                                          U   G A        C G      AUUU...ACC-5'
             |                                                                                                                                                        |
            160                                                                                                                                                      14
NEAT1:18:WT:4300-4500

interaction energy = -135.841 kcal/mol

CD36
         49417                                                                                                                                              49550
             |                                                                                                                                                 |
5'-AGA...UAUA  U                        A             C      C    A    U              C                  C             C  A        C            U      AAAA...AAA-3'
             CA GGCUGGGCAUGGUGGCUCAUGCCU UAAUCCUAGUACUU GGGAGG UGAG CGGG   GGAUCACUUGAG UCAGGAGUUUGAGACCAG CUGGGCAACAUGG AA ACCCCAU UCUAC  GAAUAA ACAAA
             || |||||||||::|||||||||::||| ||||||:|::|||| |||||| |||| ||||   |||||||||||| :|||:|||||||||||||| |:||||||||::| || ||||||: |||||  :||:|| |||||
             GU CCGACCCGUGUCACCGAGUGUGGA AUUAGGGUUGUGAA CCCUCC ACUC GCCC   CCUAGUGAACUC GGUCUUCAAACUCUGGUC GGCCCGUUGUGUC UU UGGGGUG AGAUG  UUUGUU UGUUU
     3'-CAAAA  U                        C             A      A        CCU            A                  A             U  C        U     AU             AUUU...ACA-5'
             |                                                                                                                                                 |
            195                                                                                                                                               60
NEAT1:18:WT:3000-3200

interaction energy = -116.98 kcal/mol

CD36
         33211                                                                                                                                                           33359
             |                                                                                                                                                              |
5'-AGA...UACA  A   A     CA                 U                A                   A   ACUC                     AA          CA                           A                AGCC...AAA-3'
             GGC UGC CCACCA  CCUGGCUAAUUUUUGUA UUUUAGUAGAGA GG GGUUUCACCAUGUUGGUCA GCU    UCGAACUCCUGACCUC  GUGAUCCGCC  CCUCAGCCUCCCAAAGUGCUGGGAUU CAGGUGUGAGCCACCA
             ||| ||| ||||||  ||||||||||||||||| |||||||||||| || |||:|||||||:||||:|| |||    ||||||||||||||||  |||||||:||  ||||||||||||||||||||::|||| ||||:|:|||||||||
             CCG ACG GGUGGU  GGACCGAUUAAAAACAU AAAAUCAUCUCU CC CCAGAGUGGUAUAACCGGU CGA    AGCUUGAGGACUGGAG  CACUAGGUGG  GGAGUCGGAGGGUUUCACGAUUCUAA GUCCGCGCUCGGUGGU
3'-CUC...AUGU  C   C     AC                 C            A                       C   CCAU                          AC                                                   AUGA...ACC-5'
             |                                                                                                                                                              |
            183                                                                                                                                                            38
NEAT1:18:Mut:4300-4500

interaction energy = -148.051 kcal/mol

CD36
         33211                                                                                                                                                           33359
             |                                                                                                                                                              |
5'-AGA...UACA  A   A     CA                 U                A      C            A   ACUCUC                   AA          CA                           A                AGCC...AAA-3'
             GGC UGC CCACCA  CCUGGCUAAUUUUUGUA UUUUAGUAGAGA GG GGUUU ACCAUGUUGGUCA GCU      GAACUCCUGACCUC  GUGAUCCGCC  CCUCAGCCUCCCAAAGUGCUGGGAUU CAGGUGUGAGCCACCA
             ||| ||| ||||||  ||||||||||||||||| |||||||||||| || ||||| |||||:||||:|| |||      ||||||||||||||  |||||||:||  ||||||||||||||||||||::|||| ||||:|||||||||||
             CCG ACG GGUGGU  GGACCGAUUAAAAACAU AAAAUCAUCUCU CC CCAAA UGGUAUAACCGGU CGA      CUUGAGGACUGGAG  CACUAGGUGG  GGAGUCGGAGGGUUUCACGAUUCUAA GUCCGCACUCGGUGGU
3'-CUC...AUGU  C   C     AC                 C            A      A            C   CCAUAA                        AC                                                   AUGA...ACC-5'
             |                                                                                                                                                              |
            183                                                                                                                                                            38
NEAT1:18:WT:4300-4500
```
